# Gut microbiota-metabolite interactions in drug-induced liver injury: mechanisms, biomarkers, and therapeutic perspectives

**DOI:** 10.3389/fcimb.2025.1737234

**Published:** 2025-12-15

**Authors:** Xiaoya Mao, Xujiao Hu, Jingjing Fang

**Affiliations:** 1 Department of Pharmacy, The Affiliated People's Hospital of Ningbo University, Ningbo, China; 2 Department of Clinical Laboratory, The Affiliated People’s Hospital of Ningbo University, Ningbo, China; 3 Department of Hepatobiliary Surgery, The Affiliated People’s Hospital of Ningbo University, Ningbo, China

**Keywords:** drug-induced liver injury, gut microbiota, microbial metabolites, gut-liver axis, precision hepatology

## Abstract

Drug-induced liver injury (DILI) remains a major obstacle in clinical pharmacotherapy and a leading cause of acute liver failure and drug withdrawal worldwide. Conventional mechanistic models centered on hepatic xenobiotic metabolism, oxidative stress, and immune injury cannot fully account for the substantial interindividual variability and the unpredictable nature of idiosyncratic DILI. Increasing evidence shows that the gut microbiota and its metabolites critically shape hepatic susceptibility through modulation of drug metabolism, inflammatory signaling, and intestinal barrier integrity. This review summarizes current understanding of the gut–liver axis in DILI pathogenesis, with a focus on microbial enzymes such as β-glucuronidase that reactivate detoxified drug conjugates, microbial dysbiosis that disrupts bile acid homeostasis, and depletion of short chain fatty acids and indole derivatives that normally support epithelial defenses and immunologic tolerance. Drug-specific microbial patterns are discussed, including acetaminophen, amoxicillin–clavulanate, anti-tuberculosis regimens, and immune checkpoint inhibitors. We introduce the concept of metabotype-dependent hepatotoxicity, which emphasizes that individual microbial metabolic profiles influence DILI risk. Advances in metagenomics, metabolomics, and integrative multi-omics enable the identification of microbial biomarkers and functional pathways associated with DILI susceptibility. Emerging therapeutic strategies include restoration of microbial homeostasis, selective inhibition of microbial enzymes, and supplementation of hepatoprotective metabolites. Finally, we outline key challenges and future directions toward translating microbiome-based insights into clinical prediction and precision prevention of DILI. Importantly, this review integrates microbial metabolic functions with precision hepatology concepts, highlighting how metabotype-driven variability can be leveraged for individualized DILI risk assessment.

## 1 Introduction

Drug-induced liver injury (DILI) remains a major public health concern and the leading cause of acute liver failure in Western countries (Andrade et al., 2019; Björnsson and Björnsson, 2022). It accounts for a substantial proportion of drug-related morbidity and mortality and is a frequent reason for clinical trial termination as well as post-marketing drug withdrawal (Devarbhavi et al., 2021). Despite its importance, diagnosis remains highly challenging because of its broad clinical spectrum and its tendency to mimic other hepatic disorders. Traditionally, DILI is divided into two categories according to its predictability (Kobayashi et al., 2023). Intrinsic DILI is dose-dependent, reproducible across individuals, and results from the direct toxic effects of certain compounds, such as high-dose acetaminophen (APAP) (Tiwari et al., 2025). In contrast, idiosyncratic DILI (I-DILI) is rare, unpredictable, and occurs at therapeutic doses. Its pathogenesis involves multifactorial interactions among host genetic susceptibility, immune reactivity, and environmental influences. I-DILI accounts for most severe and unexpected hepatic adverse reactions and continues to pose a major obstacle to safe drug development and clinical management (Fontana et al., 2023).

Traditional mechanistic frameworks for DILI have largely emphasized xenobiotic metabolism, oxidative stress, and direct immune-mediated hepatocellular damage (Villanueva-Paz et al., 2021; Di Zeo-Sánchez et al., 2022). These classical models have provided essential insight into hepatic toxicology but fail to fully capture the pronounced interindividual variability and the low incidence of I-DILI among exposed populations. The central limitation arises from their focus on intrinsic hepatic processes, without adequately addressing the broader host-specific factors that shape susceptibility. Genetic screening, though valuable, has offered only partial explanatory power, underscoring the need for more comprehensive models that integrate metabolic, immunological, and environmental determinants of hepatic vulnerability (Yuan and Kaplowitz, 2013; Tasnim et al., 2021; Li et al., 2023).

The human gut microbiota, a highly complex and adaptable ecosystem comprising trillions of microorganisms, serves as a functional “second genome” that exerts profound influence over host physiology, immune regulation, and drug metabolism (Wu et al., 2024). This microbial consortium harbors an extensive enzymatic repertoire that complements and often surpasses hepatic Phase I and Phase II metabolic processes, thereby reshaping drug pharmacokinetics and bioavailability (Xiang et al., 2023; Martinelli and Thiele, 2024; Kaur et al., 2025). The collective metabolic activity of the microbiota, referred to as the microbial metabolome, functions as a pivotal communication axis that modulates innate immune signaling, inflammatory tone, and intestinal barrier homeostasis (Gasaly et al., 2021). Disturbances in this microbial network and its metabolite output can markedly shift the host’s susceptibility to DILI, transforming microbial composition and metabolic activity into critical determinants of hepatotoxic risk (Huang et al., 2024).

The gut ecosystem can fundamentally modulate drug toxicity, necessitating a comprehensive re-evaluation of DILI pathogenesis from an ecological perspective (Chen et al., 2015; Fontana et al., 2023). The unpredictable nature of I-DILI, characterized by its low incidence and interindividual variability, may become more explicable if the activity of key microbial enzymes or the abundance of specific microbial co-factors can be quantified and shown to remain stable within an individual’s metabolic phenotype, or metabotype (Zimmermann et al., 2019). When microbial metabolism produces measurable hepatotoxic co-factors such as lipopolysaccharide (LPS) (Fu et al., 2024), or alters drug toxicity through enzymes like β-glucuronidase (Zeng et al., 2023), these functional microbial features transform DILI risk assessment from a probabilistic event into a measurable biochemical parameter. In this context, susceptibility to I-DILI may be viewed as a quasi-intrinsic property shaped by the host-microbiota metabolic interface.

This review consolidates emerging scientific evidence elucidating the bidirectional communication between gut microbial dysbiosis, metabolite perturbations, and susceptibility to DILI along the gut-liver axis. Its central aim is to integrate current understanding of microbial-metabolite-host interactions to clarify the concept of metabotype-dependent hepatotoxicity and its mechanistic implications. By mapping these interconnected pathways, the review highlights how individual microbial and metabolic profiles can influence hepatic responses to xenobiotics, thereby paving the way for precision biomarkers and microbiota-targeted therapeutic interventions to predict, prevent, and manage DILI with greater accuracy.

## 2 The gut-liver axis in health and drug response

### 2.1 Anatomical and physiological interdependence

The liver and intestine are closely connected through an integrated anatomical, metabolic, and immunological network known as the gut-liver axis. This axis is primarily established through the portal venous circulation, which channels blood from the small and large intestines carrying nutrients, xenobiotics, and microbial products directly to the liver (Pabst et al., 2023). This unique arrangement places the liver as the first organ exposed to both beneficial microbial metabolites such as short chain fatty acids (SCFAs) and potentially harmful substances including bacterial components and unconjugated drug metabolites (Tripathi et al., 2018; Tilg et al., 2022). Functioning as a metabolic and immune barrier, the liver performs essential roles in detoxification, biotransformation, and clearance, thereby preserving systemic homeostasis and maintaining equilibrium between immune tolerance and host defense in response to gut derived signals (Kubes and Jenne, 2018).

The enterohepatic circulation of bile acids (BAs) represents one of the most tightly regulated and essential forms of metabolic cooperation between the host and the gut microbiota (Grüner and Mattner, 2021; Li et al., 2024a). The liver synthesizes primary BAs and conjugates them with taurine or glycine to facilitate lipid digestion. When these conjugated BAs reach the intestine, commensal bacteria expressing bile salt hydrolase enzymes catalyze their deconjugation, producing secondary BAs such as deoxycholic acid and lithocholic acid. These secondary BAs are subsequently reabsorbed and transported back to the liver through the portal circulation (Urdaneta and Casadesús, 2017; Duszka, 2022). This continuous cycle plays a crucial role in maintaining hepatic metabolic balance through the activation of nuclear and membrane receptors, particularly the Farnesoid X receptor (FXR) and the Takeda G-protein coupled receptor 5 (TGR5), which collectively regulate BA synthesis, lipid metabolism, and inflammatory responses within the gut-liver axis (Chiang and Ferrell, 2020; Lin et al., 2025).

### 2.2 Microbial influence on host pharmacokinetics

The ability of the gut microbiome to shape drug exposure kinetics, often referred to as the second genome governing pharmacokinetics, is central to understanding individual susceptibility to DILI. This regulatory capacity is exerted primarily through two interconnected biochemical pathways involving microbial enzymatic activity and transcriptional modulation of host xenobiotic receptors (Tsunoda et al., 2021; Burke and Li, 2025).

Microbial enzymes, particularly β-glucuronidase, interfere with host detoxification processes. During Phase II metabolism, the liver inactivates drugs or their toxic intermediates by conjugating them with glucuronic acid. When these glucuronide conjugates enter the colon, bacterial β-glucuronidase enzymes cleave the conjugation bond, regenerating the parent compound or an active metabolite that may exert hepatotoxic effects (Gao et al., 2022; Hillege et al., 2024). This reaction facilitates enterohepatic recycling, extends systemic drug exposure, and increases hepatic toxic burden, thereby altering both the effective dose and exposure duration within an individual.

In parallel, microbial metabolites serve as potent ligands for host nuclear receptors that regulate hepatic drug-metabolizing enzymes (Prakash et al., 2015). Indole derivatives produced from bacterial tryptophan metabolism, for instance, activate the aryl hydrocarbon receptor (AhR) and the pregnane X receptor (PXR), which in turn modulate the expression of cytochrome P450 enzymes such as CYP3A4 and efflux transporters such as MDR1 (Sun et al., 2020; Vyhlídalová, 2022; Haduch et al., 2023). Through these transcriptional networks, the microbiome indirectly governs hepatic detoxification capacity and influences overall drug handling. Consequently, personalized assessment of DILI risk requires functional metagenomic profiling of microbial enzymatic potential, including quantification of β-glucuronidase gene abundance, to capture the true microbial contribution to hepatotoxic susceptibility.

### 2.3 DILI pathogenesis: the dysbiosis-barrier failure-inflammation cascade

When DILI develops, the physiological harmony between the gut and the liver deteriorates, giving way to a pathological state (Huang et al., 2024). Exposure to certain drugs, particularly antibiotics such as amoxicillin-clavulanate, can trigger profound microbial dysbiosis characterized by the depletion of protective commensal species, including SCFA producers, and the overgrowth of opportunistic or pathogenic bacteria (Kesavelu and Jog, 2023). This ecological imbalance compromises intestinal barrier integrity and alters gut metabolic output.

Under healthy conditions, SCFAs are essential and serve as the principal energy source for colonocytes and play a crucial role in preserving the structure and function of tight junctions (Yue et al., 2022). Their depletion weakens mucosal defenses and increases intestinal permeability, a phenomenon often referred to as leaky gut (Camilleri, 2019). The disrupted barrier permits the translocation of microbial components, particularly LPS, a potent pathogen-associated molecular pattern derived from the outer membrane of Gram-negative bacteria such as Proteobacteria. These translocated microbial molecules enter the portal vein and reach the liver, where they activate Kupffer cells through the Toll-like receptor 4 (TLR4) signaling pathway (Sperandeo et al., 2017).

Activation of this pathway initiates the MyD88 and NF-κB cascade, leading to the production and release of proinflammatory cytokines including TNF-α, IL-1β, and IL-6 (Guijarro-Muñoz et al., 2014). The resulting inflammatory storm promotes oxidative stress, mitochondrial injury, and hepatocyte necrosis, which are key events in the progression of DILI (Andrade et al., 2019). In this context, the gut microbiota may contribute to hepatotoxicity, providing inflammatory co-factors such as LPS that lower the hepatic threshold for drug-induced cellular injury (Huang et al., 2024).

## 3 Gut microbiota alterations in DILI: clinical and preclinical evidence

DILI is consistently linked to characteristic patterns of microbial dysbiosis, with the specific alterations in gut microbial composition varying according to the pharmacological agent responsible (Fu et al., 2022; Chu et al., 2023). These drug-specific microbial signatures suggest that distinct classes of compounds exert selective pressure on the intestinal ecosystem, shaping the abundance and metabolic activity of key bacterial taxa. Understanding these differential dysbiotic patterns provides crucial insight into how individual drugs interact with the gut microbiome to influence hepatic vulnerability and the overall trajectory of liver injury.

### 3.1 General microbial shifts and clinical evidence

Both human clinical observations and preclinical animal studies of DILI reveal consistent alterations in gut microbial composition. These changes include a decline in microbial diversity, reflected by reduced alpha diversity, and by significant restructuring of community composition across subjects, reflected by altered beta diversity (Chu et al., 2023). A recurrent feature of this dysbiosis is a reduction in the Firmicutes-to-Bacteroidetes ratio, accompanied by an overrepresentation of Gram-negative bacteria, particularly members of the phylum Proteobacteria (Chu et al., 2023). This enrichment is of particular pathophysiological importance because Proteobacteria constitute the principal source of endotoxin, or LPS. The elevated presence of LPS producing taxa enhances activation of the TLR4 pathway in the liver, amplifying inflammatory responses and lowering the threshold for hepatocellular injury following drug exposure.

### 3.2 Drug-specific mechanistic links

The gut microbiome exerts a dual influence on DILI, functioning both as a mediator of detoxification and as a contributor to hepatotoxicity. This bidirectional role underscores that therapeutic strategies for DILI cannot rely on a uniform approach (Mi et al., 2022; Chu et al., 2023). Precision medicine must instead determine whether intervention should focus on inhibiting harmful microbial activities or restoring beneficial ones that have been disrupted. Defining this balance is essential for effective management, as different pharmacological agents engage distinct microbiome-dependent pathways that either mitigate or exacerbate hepatic injury. The contrasting mechanisms observed with several high-risk drugs exemplify this complexity and highlight the need for tailored microbiome-targeted interventions in DILI prevention and therapy (**Table 1**).

**Table 1:** Drug-specific DILI mechanisms and microbial contributions.

Hepatotoxic drug class	Observed microbiota alteration/key enzyme	Primary mechanistic link to DILI	Pathway regulation
Acetaminophen	Increased microbial β-glucuronidase activity	Enhanced enterohepatic recirculation of toxic metabolites; Oxidative Stress	ROS/Nrf modulation
Amoxicillin-clavulanate	General dysbiosis; Probiotic depletion	Innate immune activation; Intestinal barrier disruption	NF-κB/Immune Signaling
Anti-tuberculosis drugs	Dysbiosis (e.g., decreased Bacteroides fragilis 839)	LPS translocation; Amplified inflammation	LPS/TLR/NF-κB pathway activation
Immune checkpoint inhibitors	Specific pre-treatment taxonomic signatures	Altered T-cell responses; Systemic immune dysregulation	Immune checkpoint modulation

#### 3.2.1 APAP-DILI

APAP overdose remains the most common cause of acute liver failure worldwide (Bunchorntavakul and Reddy, 2018; Liu et al., 2024). The extent of its hepatotoxicity is strongly modulated by gut microbial enzymatic activity (Wu et al., 2025). Following hepatic detoxification, APAP is conjugated to form a glucuronide metabolite, which is normally non-toxic and excreted through bile or urine. However, when this conjugated metabolite reaches the intestine, certain commensal bacteria, including specific strains of Clostridium perfringens, produce β-glucuronidase enzymes that hydrolyze the glucuronic acid bond (Sakaguchi et al., 1983). This reaction regenerates the parent compound or a reactive intermediate that re-enters the circulation, thereby promoting enterohepatic recirculation and prolonging hepatic exposure to the toxic metabolite (Yue et al., 2021). The extended exposure heightens oxidative stress and significantly exacerbates hepatocellular injury.

In contrast, some microbial activities exert protective effects against APAP toxicity. For instance, bacterial β-galactosidases can release metabolites such as daidzein, which has been shown to mitigate APAP-induced liver injury in experimental models. These opposing actions highlight the intricate balance of microbial influences on xenobiotic metabolism, illustrating how the gut microbiota can act as both a risk amplifier and a protective modulator in drug-induced hepatotoxicity (Zeng et al., 2023).

#### 3.2.2 Amoxicillin-clavulanate-DILI

AC is the antibiotic most frequently associated with DILI worldwide and is often classified with high causality scores (Román-Sagüillo et al., 2024). Its hepatotoxic potential may be associated with its capacity to disrupt the gut microbial ecosystem. By broadly suppressing commensal and probiotic species, AC induces marked dysbiosis that diminishes the abundance of key SCFA producing bacteria. The resulting depletion of metabolites such as butyrate undermines intestinal barrier integrity and weakens mucosal defense.

As the barrier becomes compromised, microbial antigens and pathogen-associated molecular patterns, including LPS, can translocate into the portal circulation. Their arrival in the liver stimulates innate immune activation and inflammatory signaling, leading to hepatocellular stress and cytotoxic injury. Thus, AC exemplifies a model of DILI in which loss of microbial protective functions, rather than the gain of toxic activity, drives hepatic vulnerability through gut barrier dysfunction and immune mediated hepatotoxicity (Ghosh et al., 2020).

#### 3.2.3 Anti-tuberculosis-DILI

ATB-DILI, most commonly arising from multidrug regimens containing isoniazid and rifampicin (HRZE), remains a major limitation to effective tuberculosis therapy. Its pathogenesis is closely associated with intestinal barrier disruption and activation of the gut-liver inflammatory axis (Li et al., 2025). Experimental studies have established the LPS-TLR4 pathway as a key driver of this process.

In preclinical models, administration of the probiotic strain Bacteroides fragilis 839 markedly alleviated HRZE-induced hepatotoxicity (Li et al., 2025). The protective effect was primarily mediated by restoration of microbial homeostasis and reinforcement of intestinal barrier integrity, which together led to a substantial reduction in circulating LPS concentrations. Lower LPS exposure attenuated hepatic activation of the TLR4-MyD88-NF-κB signaling cascade and consequently reduced the production of pro-inflammatory cytokines such as TNF-α and IL-6 (Gong et al., 2022). These findings underscore the pivotal role of the gut barrier in maintaining hepatic immune quiescence and highlight the therapeutic potential of microbiota-targeted interventions in mitigating inflammatory forms of DILI.

#### 3.2.4 Immune checkpoint inhibitors-DILI

ICIs have transformed the treatment landscape for cancer but are frequently accompanied by immune-related adverse events, including ICI-DILI (Xiang et al., 2022). The gut microbiome has emerged as a crucial determinant of both susceptibility and clinical outcome in these toxicities. Distinct microbial patterns have been associated with ICI-related hepatotoxicity, characterized by a marked depletion of beneficial SCFA producing taxa such as Lachnospiraceae, Ruminococcaceae, and Agathobacter (Biddle et al., 2013). The loss of these commensal bacteria reduces SCFA availability, weakens mucosal immune tolerance, and promotes dysregulated T cell activation, thereby amplifying hepatic immune injury. Accurate identification of these predictive microbial and metabolic biomarkers requires high-resolution sequencing approaches, with whole genome shotgun metagenomics providing greater functional and taxonomic precision than conventional 16S rRNA profiling (Zaplana et al., 2024).

Taken together, the divergent mechanisms observed across these drug classes highlight the dual microbial contribution to DILI pathogenesis. Hepatotoxicity may arise either from the amplification of harmful microbial functions, such as β-glucuronidase activity in APAP toxicity, or from the depletion of protective functions, including SCFA generation in antibiotic- and ICI-associated DILI and barrier maintenance in anti-tuberculosis DILI. Understanding this balance between detrimental and protective microbial processes is fundamental to developing targeted microbiome-based strategies for DILI prevention and management.

## 4 Microbial metabolites as mediators of DILI

**Figure 1:**
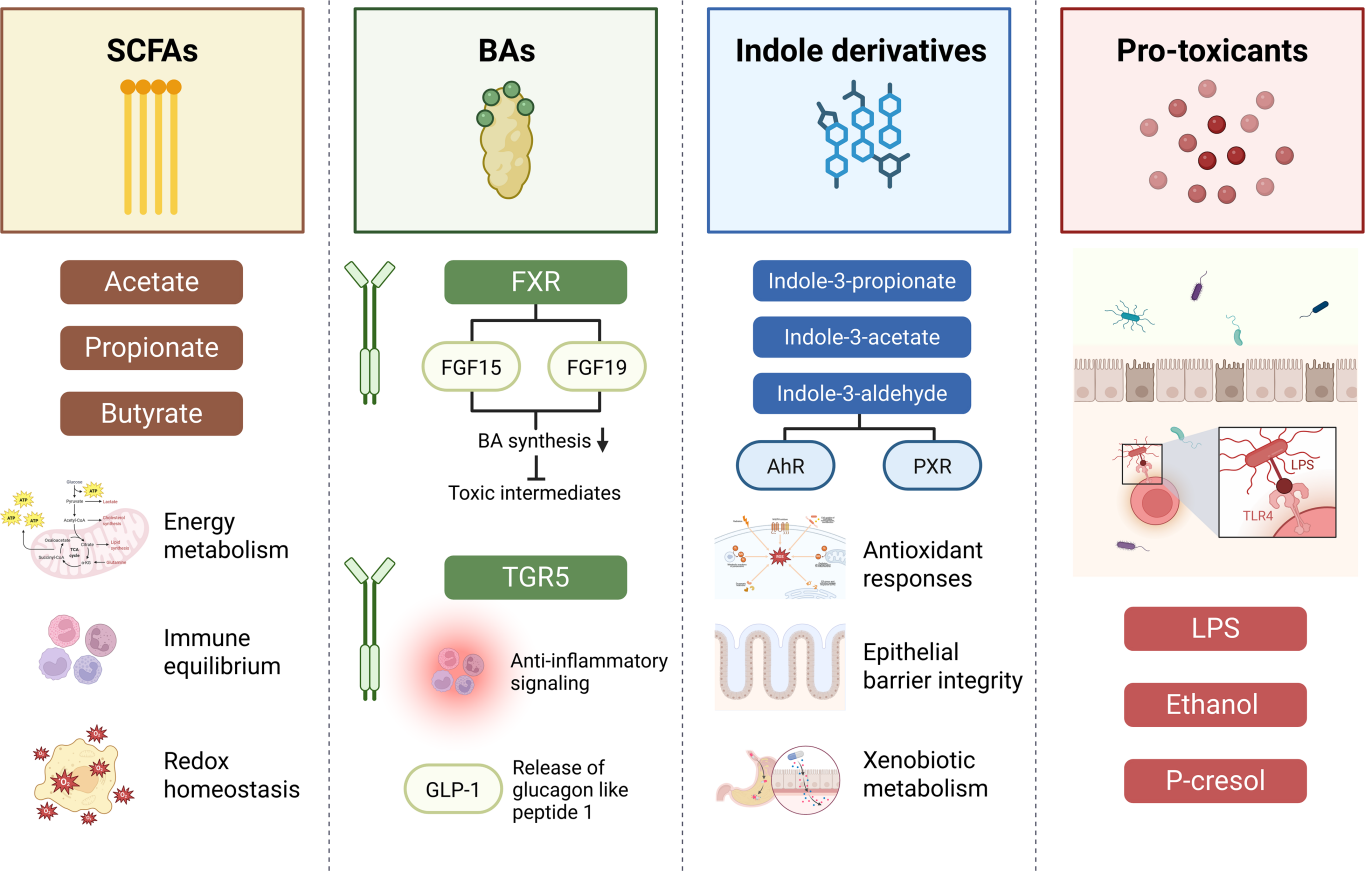
Microbiota-derived metabolites and their regulatory effects on drug-induced liver injury. SCFAs (acetate, propionate, butyrate) support energy metabolism, immune balance, and epithelial defense via GPR41/43. Bile acids modulate hepatic and intestinal signaling through FXR-FGF15/19 and TGR5. Indole derivatives (e.g., indole-3-propionate, indole-3-acetate) activate AhR/PXR to enhance antioxidant responses and barrier integrity. In contrast, pro-toxicants such as LPS, ethanol, and p-cresol activate TLR4-mediated inflammation, increasing susceptibility to DILI.

Microbial metabolites serve as the functional currency of the gut-liver axis, operating as key signaling mediators that can either confer systemic protection or intensify hepatotoxic responses (Pan et al., 2024; Tarantino et al., 2025). The susceptibility to DILI is closely influenced by the gut microbiota’s capacity to produce these bioactive compounds in adequate quantities. These metabolites act as molecular regulators that engage host metabolic and immune pathways, functioning as metabolic switchboards that fine-tune hepatic defense, detoxification, and inflammatory signaling (**Table 2**). Thus, the balance between protective and pathogenic microbial metabolite production represents a decisive factor governing individual vulnerability to DILI (**Figure 1**).

**Table 2:** Key microbial metabolites and their role in DILI protection/toxicity.

Metabolite class	Microbial precursor/source	Host receptor/target	Functional role in DILI	Outcome
SCFAs	Dietary fiber (Fermentation)	GPR41/43; HDACs	Anti-inflammatory action; Upregulation of antioxidant defense	Protection
Secondary BAs	Primary bile acids (Deconjugation)	FXR; TGR5	Regulate hepatic metabolism; Modulate inflammation (NLRP3 inflammasome)	Protection/toxicity depending on profile
Indole derivatives	Tryptophan	AhR	Activation of Phase I/II detoxification enzymes; Barrier stabilization	Protection
LPS	Gram-negative bacteria	TLR4	Activation of Kupfer cells; Release of pro-inflammatory cytokines	Toxicity

### 4.1 SCFAs

SCFAs, principally acetate, propionate, and butyrate, are produced through the fermentation of dietary fiber by anaerobic gut bacteria. They play essential roles in regulating host energy metabolism, maintaining immune equilibrium, and preserving redox homeostasis (Li et al., 2024b). A consistent feature of DILI-associated dysbiosis is the marked reduction in SCFA production, reflecting the loss of beneficial microbial functions.

The hepatoprotective effects of SCFAs operate through multiple mechanisms. Butyrate, the primary energy substrate for colonocytes, reinforces intestinal epithelial tight junctions and strengthens the mucosal barrier, thereby limiting the passage of endotoxins into the portal circulation (Yan and Ajuwon, 2017). In addition to their structural role, SCFAs act as bioactive signaling molecules by activating G protein-coupled receptors GPR41 and GPR43 and by inhibiting histone deacetylases (Kim et al., 2013). These pathways influence immune cell differentiation, enhance the generation of regulatory T cells, and suppress proinflammatory signaling cascades. Through these combined effects, SCFAs help to stabilize the gut-liver interface and mitigate immune-mediated injury, underscoring their central importance in protecting against DILI (Verma et al., 2024).

### 4.2 BAs and nuclear receptor signaling

The BA pool is continuously reshaped by the gut microbiota, exerting wide-ranging effects on host metabolic and immune signaling. Microbial bile salt hydrolase activity plays a decisive role in determining the concentration and composition of secondary BAs, which act as potent ligands for two major host receptors, FXR and TGR5 (Chiang and Ferrell, 2020; Song et al., 2025).

FXR is abundantly expressed in both the liver and intestine and serves as a central regulator of BA synthesis. When activated by BAs in the intestine, FXR induces the production of fibroblast growth factor 15 in mice or fibroblast growth factor 19 in humans. This hormone acts on the liver to suppress BA synthesis, thereby preventing the accumulation of toxic intermediates. Microbial transformations that generate secondary BAs capable of antagonizing FXR signaling, including tauro-β-muricholic acid, can disrupt this feedback mechanism, leading to dysregulated BA homeostasis and heightened susceptibility to drug-induced hepatotoxicity (Chiang et al., 2017).

In contrast, TGR5 is expressed on hepatic immune cells, including Kupffer cells, and on enteroendocrine L cells in the gut. Activation of this receptor by BAs promotes anti-inflammatory signaling within the liver and stimulates the release of glucagon like peptide 1, contributing to metabolic stability. Dysbiosis induced alterations in the BA profile weaken these protective signaling pathways and diminish hepatic resilience to toxic drug exposure, reinforcing the pivotal role of the microbiome in maintaining BA mediated liver protection (Chiang et al., 2017; Beaudoin et al., 2023).

### 4.3 Indole derivatives: detoxification boosters via tryptophan metabolism

Indole derivatives, including indole-3-propionate (IPA), indole-3-acetate, and indole-3-aldehyde, are key metabolites derived from the bacterial metabolism of dietary tryptophan (Hubbard et al., 2015). These compounds function as potent ligands for host nuclear receptors such as AhR and PXR. Activation of these receptors enhances the host’s intrinsic defense capacity by promoting antioxidant responses, strengthening epithelial barrier integrity, and regulating xenobiotic metabolism. Through these coordinated actions, indole derivatives establish a crucial microbiota-driven signaling pathway that protects the liver from oxidative stress and inflammation, thereby reducing susceptibility to drug-induced injury.

#### 4.3.1 AhR activation

Indole metabolites act as well-characterized agonists of the AhR, a pivotal transcription factor in maintaining intestinal and hepatic immune balance (Hubbard et al., 2015). Activation of AhR by indole compounds plays an essential role in preserving mucosal integrity by stimulating epithelial cell renewal and promoting goblet cell differentiation, which reinforces the protective mucus barrier. Equally important, AhR signaling induces the production of the anti-inflammatory cytokine IL-10 in immune cells, thereby limiting excessive immune activation within the gut lamina propria and preventing systemic inflammatory spillover that contributes to DILI (Zhu et al., 2018). In addition to its immunomodulatory effects, AhR activation enhances the transcription of host detoxification enzymes such as Cyp1a1, strengthening hepatic xenobiotic defense and mitigating oxidative stress. Together, these mechanisms highlight the central role of microbiota-derived indoles in sustaining gut-liver immune homeostasis and reducing susceptibility to hepatotoxic insults.

#### 4.3.2 PXR activation

IPA functions as a key microbial ligand for the PXR (Dutta et al., 2022), a central regulator of xenobiotic metabolism and intestinal barrier integrity (Dvořák et al., 2020). Activation of PXR by IPA induces the expression of major detoxification and efflux genes, including CYP3A4 and MDR1, thereby enhancing the metabolic and excretory capacity of both the liver and the intestinal epithelium. Through these coordinated actions, IPA strengthens the host’s ability to eliminate potentially harmful compounds and maintain mucosal defense. In addition to its metabolic role, IPA exerts potent anti-inflammatory effects by downregulating the expression of enterocyte-derived cytokines and suppressing activation of the NF-κB pathway. This modulation reduces endotoxin translocation and mitigates systemic inflammation, highlighting IPA as a key microbiota-derived metabolite that reinforces gut–liver homeostasis and protects against drug-induced hepatic injury.

### 4.4 Microbiome-derived pro-toxicants and the metabotype

The harmful effects of the gut microbiome in DILI arise mainly from the production and translocation of proinflammatory microbial compounds. Among these, LPS is the best characterized toxin that activates hepatic TLR4 signaling and induces acute inflammatory responses (Soares and Pimentel-Nunes, 2010; Chen et al., 2021). High circulating levels of LPS reflect a toxic metabolic state associated with immune activation and hepatic stress. Other microbial metabolites, such as ethanol and p-cresol, can also aggravate oxidative damage and contribute to mitochondrial dysfunction, thereby amplifying hepatic injury (Huang et al., 2024).

These mechanisms may underlie metabotype-dependent hepatotoxicity, which proposes that DILI risk is determined by an individual’s prevailing metabolic environment, referred to as the metabotype (Iruzubieta et al., 2015). A high risk metabotype is defined by low concentrations of protective metabolites such as SCFAs, indole derivatives, and BAs that activate the FXR, together with elevated levels of proinflammatory microbial products such as LPS and increased β-glucuronidase activity. Understanding and quantifying this metabolite profile provides a molecular framework for explaining the wide variability in idiosyncratic DILI susceptibility and offers a path toward individualized prediction of hepatotoxic risk.

## 5 Diagnostic and therapeutic implications

**Figure 2:**
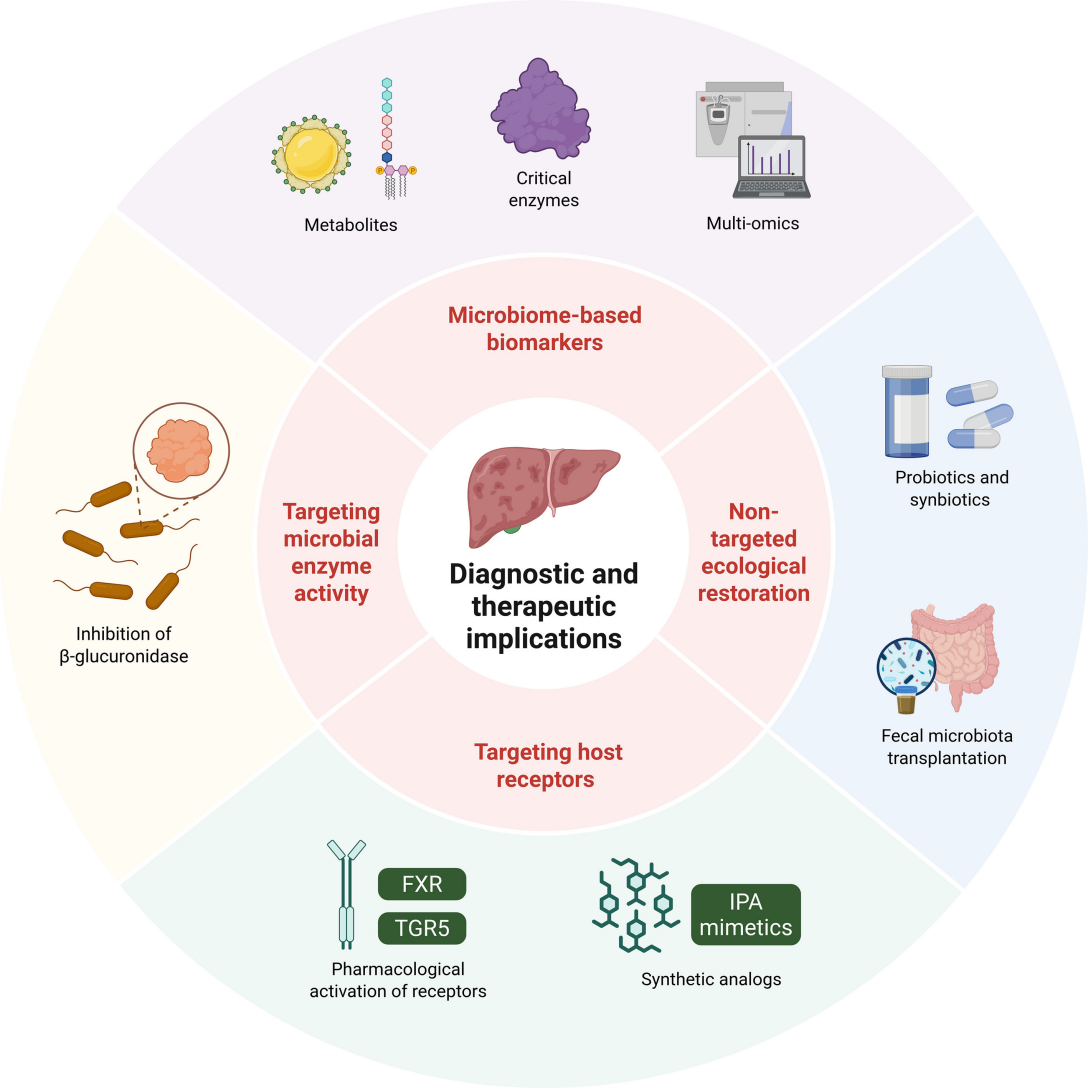
Microbiome-based diagnostic strategies and therapeutic interventions for drug-induced liver injury. Biomarker development integrates metabolites, microbial enzymes (e.g., β-glucuronidase), and multi-omics profiling. Therapeutic approaches include non-targeted ecological restoration (probiotics, synbiotics, FMT), selective inhibition of microbial enzymes, and targeting host receptors such as FXR, TGR5, or using indole-derived IPA mimetics. Together, these strategies highlight the translational potential of microbiome-guided precision hepatology.

Recognizing the gut microbiota and its metabolome as central regulators of hepatic response opens promising opportunities for advancing the diagnosis and treatment of DILI (Niu and Chen, 2020). This understanding establishes a foundation for developing next-generation diagnostic tools capable of identifying individuals at heightened risk through functional microbial and metabolic profiling (Roth et al., 2020). It also supports the design of precision therapeutic strategies that modulate specific microbial pathways or restore beneficial metabolites to strengthen hepatic resilience and prevent toxicity (**Figure 2**).

### 5.1 Microbiome-based biomarkers for personalized prediction

Current approaches to diagnosing DILI rely largely on exclusion and conventional biochemical testing, which often identify injury only after hepatic damage has occurred. Incorporating microbial and metabolic information offers a path toward proactive and individualized risk prediction (Segovia-Zafra et al., 2021).

Fecal and serum metabolite profiling provides a dynamic and noninvasive window into the metabolic communication between the gut and the liver. Measuring circulating levels of key protective metabolites such as IPA and indole-3-acetate, together with harmful compounds including secondary BAs and LPS, enables a quantifiable assessment of hepatotoxic risk (Zhang et al., 2022). In parallel, functional characterization of the gut microbiome through evaluation of gene abundance for critical enzymes such as microbial β-glucuronidase and bile salt hydrolase variants can help estimate an individual’s capacity for toxic drug reactivation or impaired detoxification (Feng et al., 2020).

Integrative multi-omics analysis is essential for capturing the full complexity of host-microbe interactions (Wang et al., 2019). Combining metagenomic, metabolomic, and transcriptomic data with clinical parameters provides the multidimensional information needed to construct robust predictive models (Sanches et al., 2024). By linking microbial functional genes with metabolite outputs and corresponding host inflammatory or detoxification gene expression patterns, researchers can identify microbial metabolic pathways that define individual susceptibility. Such integrative profiling enables advanced risk stratification, allowing clinicians to predict the likelihood of idiosyncratic DILI before drug administration and to move from reactive diagnosis toward preventive precision medicine.

### 5.2 Therapeutic modulation strategies

The dual role of the gut microbiome in DILI, functioning both as a facilitator of detoxification and as a source of proinflammatory or toxic factors, defines two principal therapeutic directions. The first involves ecological restoration, aiming to reestablish a balanced microbial community and recover lost protective functions. The second focuses on targeted inhibition, seeking to suppress specific microbial activities that contribute to hepatotoxicity (Andrade et al., 2019). Together, these complementary strategies form the foundation for microbiome-based precision therapy designed to restore gut–liver homeostasis and reduce the risk of DILI.

#### 5.2.1 Non-targeted ecological restoration

Restorative therapeutic approaches focus on reestablishing gut homeostasis and strengthening intestinal barrier integrity. Probiotics and synbiotics are designed to introduce beneficial microbial strains that promote eubiosis, enhance SCFA production, and reinforce epithelial defense (Li et al., 2022). Experimental studies have shown that Akkermansia species can alleviate APAP induced liver injury by regulating SCFA metabolism (Xia et al., 2022), while specific strains such as Lactobacillus casei and Bacteroides fragilis 839 reduce anti tuberculosis drug-induced hepatotoxicity by improving barrier function and suppressing activation of the LPS and TLR-4 signaling pathway (Li et al., 2025).

Fecal microbiota transplantation (FMT) represents a more comprehensive strategy for ecological restoration. It has proven highly effective in reconstituting microbial diversity and functional stability in conditions such as recurrent Clostridioides difficile infection (Yoon et al., 2020). In experimental models of DILI, FMT has demonstrated substantial hepatoprotective effects by rapidly restoring microbial diversity and the abundance of protective metabolites (Liu et al., 2023). Although clinical use in DILI remains limited, FMT holds considerable potential as a therapeutic option for severe or refractory cases that require rapid recalibration of the gut microbial ecosystem.

#### 5.2.2 Precision targeting of microbial enzyme activity

A chemically defined and highly targeted therapeutic strategy focuses on specific microbial functions that drive the formation of hepatotoxic intermediates, thereby eliminating the need for broad ecological modification. By concentrating on discrete enzymatic reactions, this approach simplifies intervention while maximizing precision and efficacy, particularly when the mechanism of DILI is attributable to a single microbial pathway (Huang et al., 2024).

Selective inhibition of bacterial β-glucuronidase illustrates this principle. Microbial β-glucuronidase catalyzes the reactivation of many detoxified drug conjugates, including APAP metabolites, leading to renewed hepatotoxic potential (Awolade et al., 2019). Administering selective inhibitors of this enzyme alongside the parent drug effectively prevents deconjugation, maintaining the host’s natural detoxification processes while eliminating microbial interference. Unlike antibiotic therapy, this targeted strategy avoids global disruption of the gut microbiota. Such precise enzymatic inhibition offers a promising and mechanistically grounded approach for preventing DILI, particularly for novel therapeutic agents with known susceptibility to microbial reactivation.

#### 5.2.3 Targeting host receptors via microbial metabolite analogs

A third therapeutic approach focuses on restoring or augmenting protective microbial metabolites that support hepatic resilience. This strategy involves either direct supplementation of beneficial metabolites or the use of synthetic analogs designed to mimic their physiological functions and activate host defense pathways (Mafe and Büsselberg, 2025). Pharmacological activation of receptors such as FXR and TGR5 through natural or synthetic agonists can reinforce bile acid signaling, enhance cytochrome P450 activity, and stabilize intestinal barrier integrity. Similarly, administration of stable analogs of indole derivatives, such as IPA mimetics, can stimulate the production of the anti-inflammatory cytokine IL-10 and strengthen mucosal immunity (Baars et al., 2015). These interventions offer a means to restore hepatoprotective signaling regardless of the patient’s underlying microbial composition, thereby providing a direct and controllable route to prevent or attenuate DILI.

## 6 Challenges and future directions

Despite significant advances establishing the role of the gut-liver axis in DILI, substantial challenges remain in translating these findings into clinical practice. Addressing these issues requires innovation in experimental modeling and standardization across clinical cohorts.

### 6.1 Addressing biological and clinical heterogeneity

DILI exhibits marked heterogeneity arising from the interplay of environmental, genetic, and microbial factors (García-Cortés et al., 2023). The clinical presentation and severity of DILI vary widely depending on the specific drug, patient characteristics, and underlying health status. Accounting for host variables such as metabolic disorders, including nonalcoholic fatty liver disease, or genetic predispositions, such as particular human leukocyte antigen alleles, is essential for accurately defining an individual’s susceptibility threshold. The additional influence of drug-drug interactions further complicates prediction, as concomitant therapies, including chemotherapeutic regimens in immune checkpoint inhibitor–associated DILI, can profoundly alter gut microbial composition and metabolic output, thereby modifying hepatotoxic risk.

Equally important is the recognition of racial and ethnic variability in DILI incidence and vulnerability (Lisboa et al., 2020). Baseline differences in microbiome structure, genetic background, and environmental exposures contribute to population-level disparities that remain underrepresented in existing research. Many retrospective studies fail to include sufficiently diverse cohorts, limiting the generalizability of their conclusions. Comprehensive, multi-center investigations incorporating diverse populations are therefore necessary to capture these variations and to identify groups at elevated risk, such as those with higher prevalence of metabolic disorders or distinct genetic profiles, ensuring that future DILI risk models reflect global population diversity and clinical reality.

### 6.2 Establishing causality and mechanism validation

A major limitation of current research on the gut microbiome in DILI is that most evidence remains observational and correlational (Huang et al., 2024). Although dysbiosis frequently accompanies hepatic injury, it has not been definitively established as a causal factor. Addressing this gap requires rigorous experimental validation to distinguish microbial association from mechanistic contribution.

The application of gnotobiotic animal models, in which germ-free hosts are colonized with defined microbial communities, provides a powerful tool to test causality. Similarly, targeted manipulation through selective antibiotic depletion or microbial reconstitution enables researchers to determine whether the presence or absence of specific microbial taxa or functional genes directly modifies DILI susceptibility. By demonstrating that the controlled introduction or elimination of a single microbial component can alter hepatic response to drugs, these models offer conclusive evidence for microbial causation and represent a critical step toward translating microbiome research into predictive and therapeutic applications.

### 6.3 Advanced experimental platforms for precision DILI modeling

Reproducing the complex multi-organ interactions that underlie DILI requires experimental systems capable of integrating both hepatic and intestinal physiology. Advanced *in vitro* and ex vivo platforms are emerging as indispensable tools for mechanistic validation and for evaluating individual patient susceptibility.

Human liver organoids derived from patient-specific stem cells enable high-throughput screening of intrinsic hepatotoxic potential while capturing interindividual genetic diversity (Han et al., 2024). These models provide valuable insight into direct drug cytotoxicity but remain limited in representing the systemic interactions that characterize DILI. Because DILI often results from gut-derived inflammatory mediators such as LPS, single-organ liver cultures cannot replicate the essential transport and activation processes that drive injury.

Microfluidic gut-liver chips, also known as organ-on-a-chip systems, are closing this gap (Palasantzas et al., 2023). These platforms physically and functionally connect an intestinal epithelial barrier, which can be colonized with commensal microbiota, to a hepatic module containing metabolically active hepatocytes and Kupffer cells. By allowing controlled transport of microbial metabolites and endotoxins between the gut and liver compartments, these chips faithfully reproduce the dynamic crosstalk responsible for DILI pathogenesis. Such integrative systems provide a predictive preclinical model for evaluating novel therapeutics and hold promise for personalized toxicological assessment that accounts for an individual’s unique microbial and metabolic profile.

### 6.4 Forward outlook: clinical decision support systems

The integration of microbiome, metabolome, and host data creates the foundation for developing clinically useful models that can stratify the risk of DILI. By combining pretreatment metagenomic and metabolomic profiles with genetic and clinical information, researchers can build predictive algorithms that estimate an individual’s likelihood of developing hepatotoxicity. This approach transforms DILI management from a reactive process into one centered on prevention and personalized assessment.

Such predictive systems can support clinical decision making by enabling proactive microbial interventions, including targeted enzyme inhibition or the administration of specific probiotics and prebiotics, and by guiding individualized drug dose adjustments based on predicted susceptibility (Yan et al., 2022). These developments mark a transition toward precision medicine in drug safety, where therapeutic strategies are tailored to each patient’s genetic, microbial, and metabolic characteristics to minimize the risk of hepatic injury.

## 7 Conclusions

The gut microbiota is now recognized as a fundamental and dynamic determinant of DILI, acting through continuous bidirectional communication with the liver. This microbial ecosystem maintains a delicate equilibrium, serving both protective and harmful roles. On one hand, it supports host defense by generating metabolites such as SCFAs and indole derivatives that activate the AhR and PXR. On the other hand, it can contribute to vulnerability through LPS translocation and the enzymatic reactivation of toxic metabolites, as observed with β-glucuronidase activity in APAP-related DILI.

The long-standing unpredictability of idiosyncratic DILI is increasingly being explained through measurable aspects of personalized microbial ecology, encapsulated in the concept of metabotype dependent hepatotoxicity. By identifying and quantifying microbial functions and metabolite patterns that influence drug pharmacokinetics and inflammatory thresholds, such as activation of the LPS/TLR4 pathway, researchers are transforming I-DILI from a random occurrence into a pharmacologically predictable and potentially modifiable risk.

The future of DILI prevention depends on uniting clinical pharmacology, microbial ecology, and systems biology. This integration requires the adoption of standardized multi omics protocols for risk assessment and the use of advanced experimental models, including microfluidic gut liver chips, to confirm causal relationships between microbial alterations and hepatic injury. The ultimate goal is to enable individualized intervention strategies, through targeted enzyme inhibitors or precision microbiome therapies, that enhance drug safety and usher in a new era of personalized hepatology. Overall, integrating microbial, metabolic, and genetic information may enable clinically actionable prediction models that support personalized hepatotoxicity prevention.
